# A Drug-Induced Acute Pancreatitis Retrospective Study

**DOI:** 10.1155/2020/1516493

**Published:** 2020-11-03

**Authors:** Ann-Lorie Gagnon, Alexandre Lavoie, Marie-Pier Frigon, Alban Michaud-Herbst, Karine Tremblay

**Affiliations:** ^1^Community Genomic Medicine Center, Department of Medicine, Université de Montréal and ECOGENE-21 biocluster, Chicoutimi, QC, Canada; ^2^Research Center, Centre Intégré Universitaire de Santé et de Services Sociaux du Saguenay-Lac-St-Jean, Chicoutimi, QC, Canada; ^3^Department of Pharmacy, Centre Intégré Universitaire de Santé et de Services Sociaux du Saguenay-Lac-St-Jean, Chicoutimi, QC, Canada; ^4^Department of Gastroenterology, Centre Intégré Universitaire de Santé et de Services Sociaux du Saguenay-Lac-St-Jean, Chicoutimi, QC, Canada; ^5^Department of Pharmacology-Physiology, Université de Sherbrooke, Saguenay Site, Chicoutimi, QC, Canada

## Abstract

**Background and Aims:**

Drugs are considered a relatively rare and understudied cause of acute pancreatitis (AP). The lack of convincing and conclusive data on drug-induced AP (DIAP) complicates the diagnosis as well as the identification of the causative drug. The aim of this study is to document causes of DIAP cases that occurred in the Saguenay-Lac-Saint-Jean (SLSJ) population.

**Methods:**

We have conducted a retrospective and descriptive population-based study of DIAP cases that occurred between 2006 and 2014 in the six hospitals serving the entire SLSJ population. Cases were selected from the Quebec Ministry of Health hospitalizations registry (MED-ECHO) administrative public database. A medical chart review was performed in an attempt to characterize DIAP hospitalizations and to identify the imputable drugs.

**Results:**

During the studied period, 75 cases (30.7% male, 69.3% female) were included totaling 90 hospitalizations for DIAP. Among them, 50 causative drugs were identified and were distributed in 17 different drug classes. Recurrent DIAPs were documented in 13 cases, and among them, 6 cases have experimented a positive rechallenge. Six drugs (5-fluorouracil, atorvastatin, bortezomib, nilotinib, rosuvastatin, and triamcinolone) were associated with the highest degree of evidence. The most common causative drugs of DIAP hospitalization were azathioprine (*n* = 7), followed by atorvastatin (*n* = 6), hydrochlorothiazide (*n* = 5), rosuvastatin (*n* = 4), and codeine (*n* = 4).

**Conclusions:**

This study has added new evidences about potentially pancreatitis-associated drugs in literature. This is the first study to report definite 5-fluorouracil- and triamcinolone-induced AP. An updated version of the evidence-based literature review is needed to support the clinicians in the identification of the causative drugs.

## 1. Introduction

Acute pancreatitis (AP) is characterized by sudden acute abdominal pain and a clinical course that differs greatly from one individual to another. In the United States, this inflammatory condition of the pancreas is still a leading cause of hospitalizations due to gastrointestinal diseases with an annual incidence that increased from 65.4 per 100,000 cases in 2001 to 81.9 per 100,000 in 2014 [1]. AP mortality rate varies depending on the severity of the episode reaching approximately 5% in moderate cases, and up to 40% in severe cases [2]. It is estimated that 15,000 individuals receive their first AP diagnosis each year in Canada and of these, 300 will die from its complications [3]. Moreover, about 20% of the *de novo* AP cases will experience recurrent acute pancreatitis (RAP), defined by at least two separate episodes with a period of resolution in between [4]. It is well known that the accumulation of AP crises can lead to an irreversible damage to the pancreas, which can ultimately alter pancreatic functions [4]. Consequently, the identification of AP etiology appears essential in order to manage the trigger, to improve AP outcomes, and to prevent RAP episodes. Heavy alcohol consumption and gallstones are the most frequent causes of AP, accounting for approximately 60% of the cases [5]. However, less frequent causes are also responsible for various forms of AP [6]. Among them, drugs are a rare cause of AP and account for approximately 0.1% to 5.0% of all cases [7–9]. The prevalence of drug-induced AP (DIAP) may be underestimated since the diagnosis is often complex as there are no unique clinical features that distinguish this etiology from others [7, 10]. Generally, DIAP is suspected once all other causes have been excluded and when there is a reasonable time sequence between the drug administration and the AP onset. In spite of the consequences on the patients' quality of life, the diagnosis is only established when the drug leading to the AP symptoms is reintroduced (called a rechallenge) [11]. In 2007, Badalov and colleagues performed a systematic review on potential drugs associated with AP and suggested a classification system divided into four categories based on the published weight of evidence [12]. To date, more than 500 drugs have been acknowledged as potential causes of AP [13]. The majority of those have been reported only under case reports or case-control studies with a low level of evidence [13].

The lack of convincing and conclusive data on DIAP complicates its diagnosis as well as the identification of the causative drug in order to avoid recurrence. Thus, we postulated that documenting the DIAP cases of the Saguenay-Lac-Saint-Jean (SLSJ) region (Quebec, Canada) will add new evidences on causative drugs in addition to providing an overall picture of the etiological characteristics of this health condition. The aim of the present study is to report DIAP cases observed in the SLSJ population.

## 2. Materials and Methods

### 2.1. Study Design and Identification of Cases

We have conducted a retrospective and descriptive population-based study of DIAP cases that occurred in the SLSJ hospitals between April 2006 and December 2014 (a total of six hospitals serving the entire SLSJ population; *n* = 277,141 individuals in 2017) [14]. The SLSJ is a French-Canadian founder population [15]. Data were extracted from the administrative Quebec Ministry of Health hospitalizations registry (MED-ECHO) by a hospital medical archivist. The MED-ECHO database provides data on acute care hospital admissions for patients covered by the Quebec public health insurance plan. All hospitalization events reported in MED-ECHO are classified according to the *International Classification of Diseases,* 10th *revision* codes [16]. Code K85.3 “Drug Induced Acute Pancreatitis” has been used to select cases and to extract relevant data. In order to ensure confidentiality, an anonymization number was attributed to each identified case. This study has been approved by the institutional ethic review board.

### 2.2. Medical Charts Review

**Figure 1:**
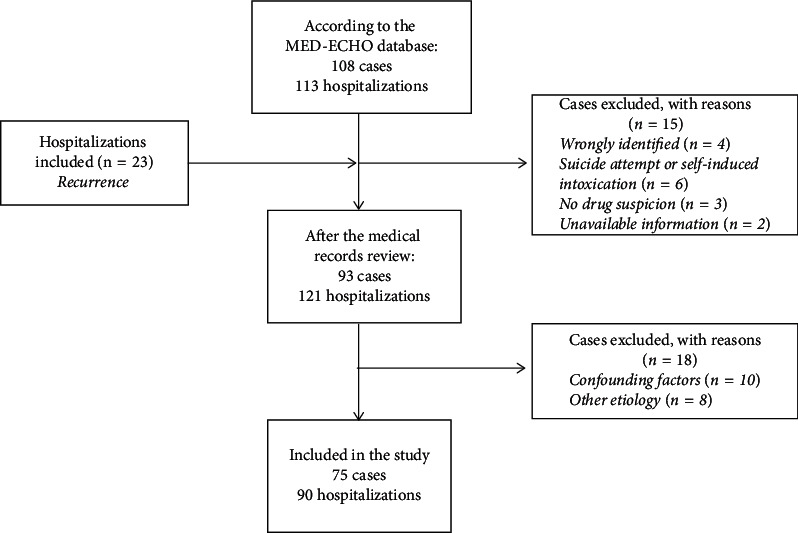
Schematic representation of the data sorting process. Flow chart representing the number of cases included in the study.

The retrospective characterization of DIAP hospitalizations has been performed by a medical chart review, and data have been manually collected in individualized paper case report forms. Demographics, anthropometrics, lifestyle habits, and comorbidities were the variables collected in order to get the most accurate clinical profile. Assessment of alcohol consumption has been done according to the Canada's Low-Risk Alcohol Drinking Guidelines [17], and cases were divided into three categories (none, active, and former alcohol consumers). The active alcohol consumption category includes men and women who drank less than three drinks a day [17]. Drinking three drinks and more a day was considered as an exclusion criterion due to the possibility of a confounding effect. Assessment of tobacco consumption was divided into the same categories, except for the frequency distinction that has not been taken into consideration due to information not being available. AP was diagnosed by treating physicians according to the Atlanta's classification criteria [18]. For each confirmed DIAP, available information on diagnosis, trigger, symptoms, treatments and interventions has been collected. The pharmacological profile at the time of admission and information on drugs' posology, duration, and indication were also collected. The drugs suspected and confirmed as causative by the treating physicians were deemed as the causative drugs for the study. Our data sorting process is presented in Figure 1. DIAP hospitalizations that have occurred outside of the studied period were included in order to document the AP recurrence. On the other hand, cases whose treating physicians did not suspect any medication during the hospitalization and cases with DIAP hospitalizations due to suicide attempt or self-induced intoxication were excluded. Missing data were also an exclusion criterion. All medical charts have been reviewed by a unique observer. A 10% validation of the data collected in the case report forms and of the electronic data entry has been successfully performed by an independent observer (correspondence rate of 97.5% and 99.1%, respectively).

### 2.3. Validation of Suspected Causative Drugs

The probability of an adverse drug reaction (ADR) for each suspected drug has been estimated based on the Naranjo's algorithm [19]. This easy-to-use algorithm is the main tool used by health professionals to evaluate ADR. This point system consists of ten questions and provides an ADR probability category according to the total score [19]. The Naranjo's algorithm has been applied on each drug found in the cases' pharmacological profile to confirm the suspected causative drugs (a total of 866 drugs) and a 20% test–retest validation has been performed by the study pharmacist (AL): all the suspected pancreatitis-associated drugs (*n* = 16) were appropriately identified (correspondence rate of 100%) and a correspondence rate of 94.9% has been reached on the remaining entire pharmacological profile (*n* = 158 assessed drugs). The discordant findings have been discussed and agreed.

### 2.4. Data Analysis and Interpretation

Descriptive statistics include numerical variables reported as geometric mean (with standard deviation (SD)) and median (with range) as well as categorical variables reported as number (with proportion). Thereafter, each suspected drug has been classified according to the therapeutic subgroup (2nd level) in the Anatomical Therapeutic Chemical Classification System proposed by the World Health Organization [20]. Finally, the evidence-level for pancreatitis-associated drugs was assessed and presented according to the classification system proposed by Badalov and colleagues [12].

## 3. Results

A total of 108 cases were hospitalized for DIAP in the SLSJ region during the studied period (Figure 1). On review of the medical charts, 23 additional admissions for DIAP were identified and added to the data, while 33 of the cases met the exclusion criteria (summarized in Figure 1). Overall, 75 cases totaling 90 DIAP hospitalizations were included in the study.

The characteristics of the identified cases are presented in Table 1. Briefly, the mean age at first admission was 58 years with a female predominance (70%). Approximately 17% of the cases have had recurrent DIAP, and among them, 8% have been rechallenged to the same drug. About half of the cases neither consume alcohol (45.3%; *n* = 34) nor tobacco products (50.7%; *n* = 38). High blood pressure (62.7%; *n* = 47), hypercholesterolemia (45.3%; *n* = 34), and cardiovascular diseases (41.3%; *n* = 31) were the most relevant comorbidities reported. Type 2 diabetes, endocrinal diseases, cancer, and bowel diseases were found in less than 30% of cases with DIAP. Cases with a diagnosis of pancreatic cancer before and after the DIAP hospitalization were excluded due to its confounding effect with obstructive AP etiology.

Among the total DIAP hospitalizations, the causal relationship with the suspected drug was considered “probable” in 84 cases (93.3%). See Table S1 in the Supplemental Material to see the ADR probability of each causative drug assessed by the Naranjo's algorithm.  Table 2 shows the principal characteristics of the DIAP hospitalizations, including laboratory values when available. The mean inhospital length of stay was 7 days (±8 days). The treating physicians have also identified three severe DIAP cases (assessed with standard Modified Marshall Scoring system based on oxygen saturation, serum creatinine, and systolic blood pressure) that required intensive care unit (ICU) support during their hospitalization. However, severity assessments were not reported by the physicians for the other cases and were not performed by our research team since such data were not available in the medical chart, unless patients were admitted to an ICU. Interestingly, the median number of drugs used by the cases indicates that most of them had polypharmacy (10 drugs and more) [21]. Lipase level was more than three times over the normal ranges, confirming a diagnosis of AP. The triglycerides and total calcium median values were found to be within the normal ranges, which prove that these two potential AP causes have not been included.

**Figure 2:**
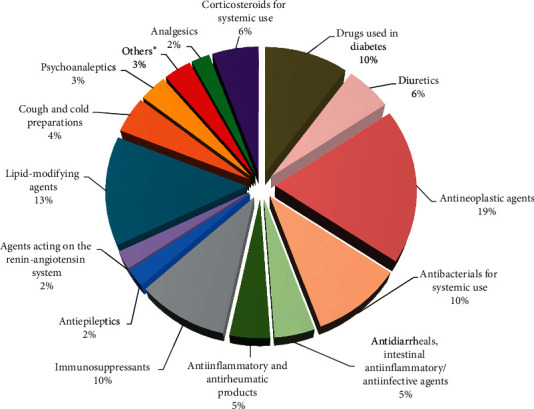
Distribution of drug-induced acute pancreatitis causative drug classes. Presented as percent according to the number of hospitalizations (*n* = 90). ^*∗*^Others included psycholeptics, endocrine therapy, and antimycobacterials drug classes.

The 51 DIAP identified causative or potentially causative drugs (available in Supplementary Table S1) are distributed across 17 different classes (Figure 2). The most frequent drug classes were antineoplastic agents (19%) and lipid-modifying agents (13%), followed by antibacterial for systemic use (10%), immunosuppressants (10%), and drugs used for diabetes (10%).

According to the evidence-level classification system [12], six imputable drugs (5-fluorouracil, atorvastatin, bortezomib, nilotinib, rosuvastatin, and triamcinolone) were associated with the highest degree of evidence (Class Ia, Table 3). In our study, no drug was found in class Ib since potential causes of AP (e.g., heavy alcohol consumption, gallstones, hypertriglyceridemia, and hyperkalemia) were ruled out. Three drugs having at least four reported cases of pancreatitis are found in class II (Table 3). However, the latency period was not taken into account for this class since in some cases, not enough data were available (e.g., drug's start and end dates). Class III shows the ten drugs responsible for at least two pancreatitis-associated hospitalizations, and the remaining drugs (*n* = 32) were classified in class IV (one pancreatitis-associated hospitalization). Finally, five of the identified drugs (azathioprine, atorvastatin, hydrochlorothiazide, rosuvastatin, and codeine) represent the most common drugs associated with DIAP in our retrospective study. These drugs are responsible for nearly 30% of all hospitalizations.

## 4. Discussion

Although drugs are a known cause of AP, the associated risk of causative drugs remains unclear since information about this AP form remains scarce [11]. The present study reports the first data on the etiological characteristics of DIAP hospitalizations that occurred in the SLSJ population. Our results show that drugs are a relatively rare cause of AP in this population (0.03%) which is similar to the findings of previous studies made in Korea (0.05%) [22], France (0.2%) [23], and Switzerland (0.3%) [24]. Moreover, a prior study in the same population using the MED-ECHO database reported 1610 AP hospitalizations that have occurred in the same study period [25], among which our DIAP cases represent 2.7%, a proportion similar to what is observed in the literature [7–9].

The demographic data show that twice as many women were hospitalized with an overall mean age of 58 years. In fact, it has been reported that the development of DIAP occurs more often in some specific patient population such as women [8, 9, 26] and elderly patients with polypharmacy [27]. Our results are consistent with these observations.

We have also highlighted six cases that have experienced positive rechallenge for four imputable drugs known to be associated with AP. Among them, one drug (bortezomib) has numerous documented cases of positive rechallenge [28–30]. In fact, it is well known that pancreatitis is a rare ADR of this antineoplastic agent. On the other hand, atorvastatin and rosuvastatin, two lipid-modifying agents, have been reported as causative agents in, respectively, six and four hospitalizations, in addition to the occurrence of a positive rechallenge. This association is also well documented [31–34]. Lai and colleagues [35, 36] observed in two independent studies an increased risk of pancreatitis in patients with current use of atorvastatin (odds ratio of 1.67) [35] and rosuvastatin (odds ratio of 3.21) [36] as compared with those who never used these drugs. In 2010, Pezzilli and colleagues [37] reported a 1% AP rate among patients using nilotinib, an antineoplastic agent. Since then, new case reports have incriminated this medication, increasing the level of evidence [38–40]. Finally, AP induced by 5-fluorouracil (an antineoplastic agent) and triamcinolone (a corticosteroid for systemic use) was unexpected since case of no positive rechallenge has been reported with these drugs. In fact, our study reports the first case of triamcinolone-induced AP.

Interestingly, azathioprine, an immunosuppressant indicated to treat inflammatory bowel diseases (IBD) and also the first-line drug for other gastrointestinal diseases such as autoimmune hepatitis [41], was the most frequent causative factor for DIAP hospitalizations in our study (*n* = 7). Azathioprine is notable for its strong association with AP and few cases of positive rechallenge have been confirmed [9, 22, 42, 43]. However, patients with IBD may be at an increased risk for AP [44]. Thus, definite DIAP diagnosis is hard to make in this patient group. Hydrochlorothiazide is another drug incriminated as the causal trigger for five DIAP hospitalizations in SLSJ. Several reports on hydrochlorothiazide-induced AP have been published [22, 43, 45] but, so far, no case of positive rechallenge has been documented. Mechanisms of azathioprine and hydrochlorothiazide in AP physiopathology are still misunderstood. Finally, even if only a small number of studies have reported codeine as the main cause of DIAP [8, 22, 26, 46], well-documented rechallenge cases do exist [46]. In our study, four DIAP hospitalizations were due to codeine. In fact, codeine is known to cause constriction of the sphincter of Oddi, which can initiate an AP episode [27, 47]. The other medications we found to be potentially causative drugs were not as frequent (*n* ≤ 3) (Table 3).

In addition to the limited data availability in some cases, there are other limitations to this study. Notably, DIAP cases could have been underrepresented. A previous study using the MED-ECHO database showed an underestimated prevalence of many chronic conditions, such as pancreatitis [48]. This can be explained by the fact that some individuals come to the emergency room but then are not hospitalized. This possible underrepresentation of DIAP cases could potentially influence the proportions of the causative drugs we have observed in the SLSJ population. However, the use of the ICD-10 codes in the MED-ECHO database allowed us to obtain data of a greater quality of than those collected with self-reported systems. Finally, polypharmacy had already been identified as a significant risk factor for DIAP [27, 49] (more than fourfold increased risk with ten or more drugs [49]). Considering that cases included in this study were taking in an average of ten drugs, AP events could have been caused by the association of numerous drugs rather than by a single one. Nevertheless, this study not only adds to the evidence on well-known pancreatitis-associated drugs but also highlights new drugs (5-fluorouracil and triamcinolone) with high level of causative evidence, which may be of interest to clinicians.

The classification of drugs proposed by Badalov and colleagues [12] is an important tool used by clinicians when DIAP is suspected since it is, so far, the only available systematic review on DIAP causative drugs. However, since 2007, many case reports documenting new DIAP causative drugs have been published. These new evidences may modify the Badalov's current classification level for some of the drugs. As an example, adding the previously described drugs to those included in Badalov's table [12], 13 drugs would now be classified with a higher level of causative evidence while 25 would be added in the table as new potentially pancreatitis-associated drugs (highlighted in bold in Table 3).

## 5. Conclusion

In conclusion, our study described new DIAP cases, adding them to the current literature, and shed light on new causative drugs with high level of evidence (5-fluorouracil and triamcinolone). To our opinion, updating the systematic review on the DIAP causative drugs following a level of evidence classification such as the one proposed by previous authors is mandatory to help clinicians who are suspecting DIAP.

## Figures and Tables

**Table 1:** Characteristics of drug-induced acute pancreatitis identified in the cases reported.

	Cases, Total *n* = 75
*Demographics*	
Male, *n* (%)	23 (30.7)
Age in years, mean (SD)^a^	58 (17.0)
Recurrent DIAP, *n* (%)	13 (17.3)
Rechallenge, *n* (%)	6 (8.0)

*Life habits, n* (%)^b^	
Alcohol consumption^c^	
None	34 (45.3)
Active	33 (44.0)
Former	3 (4.0)
Tobacco consumption	
None	38 (50.7)
Active	17 (22.7)
Former	19 (25.3)

*Relevant comorbidities, n (%)*	
High blood pressure	47 (62.7)
Hypercholesterolemia	34 (45.3)
Cardiovascular diseases	31 (41.3)
Type 2 diabetes	21 (28.0)
Endocrinal diseases	18 (24.0)
Cancer^d^	18 (24.0)
Bowel diseases	12 (16.0)

SD = standard deviation; DIAP = drug-induced acute pancreatitis; *n* = number. ^a^Mean age at first admission. ^b^Only if the information was available in the medical chart. ^c^Cases with heavy alcohol consumption have been removed from the study due to confounding factors. ^d^Cases with pancreatic cancer have been removed from the study due to confounding factors.

**Table 2:** Characteristics of drug-induced acute pancreatitis hospitalizations that occurred in the Saguenay-Lac-Saint-Jean hospitals.

	Hospitalization Total *n* = 90	Normal values range^a^
*Characteristics*		
Length of stay, mean (SD)	7 (8)	
ICU visit, *n* (%)	3 (3.3)	
Medication number used, median (range)	10 (2–22)	

*Lab values* (*median* (*range*))^b^		
Lipase in U/L (*n* = 89)	1010 (30–78, 762)	11–82
Amylase in U/L (*n* = 89)	139 (6–8244)	29–103
Triglycerides in mmol/L (*n* = 59)	1.3 (0.6–5.2)	0.00–2.2
Total calcium in mmol/L (*n* = 56)	2.2 (1.8–2.5)	2.2–2.6
C-reactive protein in mg/mL (*n* = 26)	51.8 (0.4–463.1)	0–10

SD = Standard deviation; ICU = Intensive care unit; *n* = number. ^a^According to the normal values' range used by the Centre intégré universitaire de santé et de services sociaux of Saguenay–Lac-Saint-Jean. ^b^Only if the information was available in the medical chart.

**Table 3:** Drug-induced acute pancreatitis causative drugs (number of hospitalizations) classified according to the evidence-level classification system proposed by Badalov and colleagues [12] (*n* = 90).

Class Ia^a^	Class Ib^b^	Class II^c^	Class III^d^	Class IV^e^	
5-Fluorouracil (2)	N/A	Azathioprine (7)	**Amoxicillin–clavulanic acid** ^*∗*^ **(2)**	**Azithromycin**	**Lurasidone**
Atorvastatin (6)		Codeine (4)	Cisplatin (3)	**Canagliflozin**	Metformin
**Bortezomib (2)**		Hydrochlorothiazide (5)	Dexamethasone (2)	Capecitabine	**Methotrexate**
**Nilotinib (2)**			Mercaptopurine (3)	Carbamazepine	Metronidazole
Rosuvastatin (4)			Mesalazine (3)	**Celecoxib**	Minocycline
**Triamcinolone (3)**			**Saxagliptin (2)**	**Certolizumab**	**Morphine**
			**Sitagliptin (2)**	Clarithromycin	Naproxen
			**Sitagliptin–metformin** ^*∗*^ **(2)**	**Clindamycin**	**Pazopanib**
			Trimethoprim–sulfamethoxazole^*∗*^(2)	Cyclophosphamide-doxorubicin^*∗*^	**Perindopril**
			**Venlafaxine (2)**	Cyclophosphamide-vincristine-doxorubicin^*∗*^	**Phenytoin**
				Diclofenac	Rifampin
				**Ezetimibe**	**Saxagliptin-metformin** ^*∗*^
				**Ibuprofen**	Sertraline
				**Immediate-release morphine**	Simvastatin **sulfasalazine**
				Losartan/hydrochlorothiazide^*∗*^	Tamoxifen
					**Tocilizumab**

^a^Class Ia included drugs for which at least one case of rechallenge has been described and all potential causes of acute pancreatitis have been excluded. ^b^Class Ib included drugs for which at least one case of rechallenge has been described and the potential causes of acute pancreatitis have not been excluded. ^c^Class II included drugs found in at least four evidences. ^d^Class III included drugs found in at least two evidences. ^e^Class IV included drugs found in at least one evidence. Each individual medication included in this class was implicated in only 1 case. The asterisk (^*∗*^) means that the drugs are in association. The drugs in bold are not present in the systematic review of Badalov et al. (2007).

## Data Availability

The case report forms which consist of anonymized paper files where medical data used for the present study and collected from the medical chart review can be made available by the corresponding author upon request. However, the archived medical charts of each case included in this study cannot be made available for consultation by others who are not part of the research team in order to protect confidentiality. Important to note is that the source-document-related and collected data are in the French language.

## Supplementary Materials

Supplementary Materials Table S1 illustrates the potentially causative drugs of acute pancreatitis (AP) in the Saguenay-Lac-Saint-Jean population. The probabilities of adverse drug reaction, assessed by the Naranjo's algorithm, are also demonstrated in the last column. Information concerning the Naranjo's algorithm is found in the Materials and Methods section.
