# Supplementary material for: A Drug-Induced Acute Pancreatitis Retrospective Study
Source: Can J Gastroenterol Hepatol. 2020 Nov 3;2020:1516493. doi: 10.1155/2020/1516493 (PMC7655261; doi:10.1155/2020/1516493)
Supplement: Supplementary Materials — Table S1 illustrates the potentially causative drugs of acute pancreatitis (AP) in the Saguenay-Lac-Saint-Jean population. The probabilities of adverse drug reaction, assessed by the Naranjo's algorithm, are also demonstrated in the last column. Information concerning the Naranjo's algorithm is found in the Materials and Methods section. [file 1516493.f1.docx]

**Supplementary Table 1. List of the Medications Found to be Causative of the Drug-Induced Acute Pancreatitis Cases**

| **ID** | **Medication** | | **Indication** | | **Naranjo Scale ^1^** | | | | | | | | | | | | | | | | | **Score** | **Outcome** |
| --- | --- | --- | --- | --- | --- | --- | --- | --- | --- | --- | --- | --- | --- | --- | --- | --- | --- | --- | --- | --- | --- | --- | --- |
|  |  |  |  |  | 1 | 2 | 3 | 4 | 5 | | | 6 | | 7 | | 8 | | 9 | | 10 | |  |  |
| ***Immunosuppressants*** | | |  | |  |  |  |  |  | | |  | |  | |  | |  | |  | |  |  |
| HOP-510 | Azathioprine | | Crohn's disease | | 1 | 2 | 1 | 0 | -1 | | | 0 | | 0 | | 1 | | 0 | | 1 | | 5 | Probable |
| HOP-516 | Azathioprine | | Crohn's disease | | 1 | 2 | 1 | 0 | -1 | | | 0 | | 0 | | 1 | | 0 | | 1 | | 5 | Probable |
| HOP-518 | Azathioprine | | Crohn's disease | | 1 | 2 | 1 | 0 | -1 | | | 0 | | 0 | | 1 | | 0 | | 1 | | 5 | Probable |
| HOP-523 | Azathioprine | | Crohn's disease | | 1 | 2 | 1 | 0 | 2 | | | 0 | | 0 | | 1 | | 0 | | 1 | | 8 | Probable |
| HOP-536 | Azathioprine | | Crohn's disease | | 1 | 2 | 1 | 0 | -1 | | | 0 | | 0 | | 1 | | 0 | | 1 | | 5 | Probable |
| HOP-586 | Azathioprine | | Crohn's disease | | 1 | 2 | 1 | 0 | -1 | | | 0 | | 0 | | 1 | | 0 | | 1 | | 5 | Probable |
| HOP-606 | Azathioprine | | Crohn's disease | | 1 | 2 | 1 | 0 | -1 | | | 0 | | 0 | | 1 | | 0 | | 1 | | 5 | Probable |
| HOP-551 | Certolizumab | | Rheumatoid arthritis | | 0 | 2 | 1 | 0 | -1 | | | 0 | | 0 | | 1 | | 0 | | 1 | | 4 | Possible |
| HOP-578 | Tocilizumab | | Rheumatoid arthritis | | 1 | 2 | 1 | 0 | -1 | | | 0 | | 0 | | 1 | | 0 | | 1 | | 5 | Probable |
| ***Drugs used in diabetes*** | | |  | |  |  |  |  |  | | |  | |  | |  | |  | |  | |  |  |
| HOP-568 | Canagliflozin | | Diabetes | | 1 | 2 | 1 | 0 | -1 | | | 0 | | 0 | | 1 | | 0 | | 1 | | 5 | Probable |
| HOP-587 | Metformin | | Diabetes | | 1 | 2 | 1 | 0 | -1 | | | 0 | | 0 | | 1 | | 0 | | 1 | | 5 | Probable |
| HOP-500 | Saxagliptin | | Diabetes | | 1 | 2 | 1 | 0 | -1 | | | 0 | | 0 | | 1 | | 1 | | 1 | | 6 | Probable |
| HOP-538 | Saxagliptin | | Diabetes | | 1 | 2 | 1 | 0 | -1 | | | 0 | | 0 | | 1 | | 0 | | 1 | | 5 | Probable |
| HOP-571 | Saxagliptin-Metformin | | Diabetes | | 1 | 2 | 1 | 0 | -1 | | | 0 | | 0 | | 1 | | 0 | | 1 | | 5 | Probable |
| HOP-568 | Sitagliptin | | Diabetes | | 1 | 2 | 1 | 0 | -1 | | | 0 | | 0 | | 1 | | 0 | | 1 | | 5 | Probable |
| HOP-587 | Sitagliptin | | Diabetes | | 1 | 2 | 1 | 0 | -1 | | | 0 | | 0 | | 1 | | 0 | | 1 | | 5 | Probable |
| HOP-500 | Sitagliptin-Metformin | | Diabetes | | 1 | 2 | 1 | 0 | -1 | | | 0 | | 0 | | 1 | | 1 | | 1 | | 6 | Probable |
| HOP-553 | Sitagliptin-Metformin | | Diabetes | | 1 | 2 | 1 | 0 | -1 | | | 0 | | 0 | | 1 | | 0 | | 1 | | 5 | Probable |
| ***Diuretics*** |  | |  | |  |  |  |  |  | | |  | |  | |  | |  | |  | |  |  |
| HOP-503 | Hydrochlorothiazide | | High blood pressure | | 1 | 2 | 1 | 0 | -1 | | | 0 | | 0 | | 1 | | 0 | | 1 | | 5 | Probable |
| HOP-509 | Hydrochlorothiazide | | High blood pressure | | 1 | 2 | 1 | 0 | -1 | | | 0 | | 0 | | 1 | | 0 | | 1 | | 5 | Probable |
| HOP-513 | Hydrochlorothiazide | | High blood pressure | | 1 | 2 | 1 | 0 | -1 | | | 0 | | 0 | | 1 | | 0 | | 1 | | 5 | Probable |
| HOP-549 | Hydrochlorothiazide | | High blood pressure | | 1 | 2 | 1 | 0 | -1 | | | 0 | | 0 | | 1 | | 0 | | 1 | | 5 | Probable |
| HOP-578 | Hydrochlorothiazide | | High blood pressure | | 1 | 2 | 1 | 0 | -1 | | | 0 | | 0 | | 1 | | 0 | | 1 | | 5 | Probable |
| ***Antineoplastic agents*** | | |  | |  |  |  |  |  | | |  | |  | |  | |  | |  | |  |  |
| HOP-504 | Bortezomib | | Multiple myeloma | | 1 | 2 | 1 | 2 | -1 | | | 0 | | 0 | | 1 | | 1 | | 1 | | 8 | Probable* |
| HOP-603 | Bortezomib | | Multiple myeloma | | 1 | 2 | 1 | 2 | -1 | | | 0 | | 0 | | 1 | | 1 | | 1 | | 8 | Probable |
| HOP-531 | Capecitabine | | Breast cancer | | 1 | 2 | 1 | 0 | -1 | | | 0 | | 0 | | 1 | | 0 | | 1 | | 5 | Probable |
| HOP-506 | Cisplatine | | Lung cancer | | 1 | 2 | 1 | 0 | -1 | | | 0 | | 0 | | 1 | | 0 | | 1 | | 5 | Probable |
| HOP-580 | Cisplatine | | Amygdala cancer | | 1 | 2 | 1 | 0 | -1 | | | 0 | | 0 | | 1 | | 0 | | 1 | | 5 | Probable |
| HOP-602 | Cisplatine | | Lung cancer | | 1 | 2 | 1 | 2 | -1 | | | 0 | | 0 | | 1 | | 1 | | 1 | | 8 | Probable |
| HOP-520 | Cyclophosphamide-Vincristine-Doxorubicin | | Non-Hodgkin's lymphoma | | 1 | 2 | 1 | 2 | -1 | | | 0 | | 0 | | 1 | | 0 | | 1 | | 7 | Probable |
| HOP-567 | Cyclophosphamide-Doxorubicin | | Breast cancer | | 1 | 2 | 1 | 0 | -1 | | | 0 | | 0 | | 1 | | 0 | | 1 | | 5 | Probable |
| HOP-515 | Mercaptopurine | | Crohn's disease | | 1 | 2 | 1 | 0 | -1 | | | 0 | | 0 | | 1 | | 0 | | 1 | | 5 | Probable |
| HOP-557 | Mercaptopurine | | Crohn's disease | | 1 | 2 | 1 | 0 | -1 | | | 0 | | 0 | | 1 | | 0 | | 1 | | 5 | Probable |
| HOP-564 | Mercaptopurine | | Crohn's disease | | 1 | 2 | 1 | 0 | -1 | | | 0 | | 0 | | 1 | | 0 | | 1 | | 5 | Probable |
| HOP-539 | Methotrexate | | Rheumatoid arthritis | | 1 | 2 | 1 | 0 | -1 | | | 0 | | 0 | | 1 | | 0 | | 1 | | 5 | Probable |
| HOP-604 | Nilotinib | | Leukemia | | 1 | 2 | 1 | 2 | -1 | | | 0 | | 0 | | 1 | | 1 | | 1 | | 8 | Probable* |
| HOP-604 | Nilotinib | | Leukemia | | 1 | 2 | 1 | 2 | -1 | | | 0 | | 0 | | 1 | | 1 | | 1 | | 8 | Probable* |
| HOP-521 | Pazopanib | | Kidney cancer | | 1 | 2 | 1 | 0 | -1 | | | 0 | | 0 | | 1 | | 0 | | 1 | | 5 | Probable |
| HOP-547 | 5-fluorouracil | | Colon cancer | | 1 | 2 | 1 | 2 | -1 | | | 0 | | 0 | | 1 | | 0 | | 1 | | 7 | Probable* |
| HOP-547 | 5-fluorouracil | | Colon cancer | | 1 | 2 | 1 | 2 | -1 | | | 0 | | 0 | | 1 | | 1 | | 1 | | 8 | Probable* |
| ***Antibacterials for systemic use*** | | |  | |  |  |  |  |  | | |  | |  | |  | |  | |  | |  |  |
| HOP-527 | Amoxicillin - clavulanic acid | | Bronchitis | | 1 | 2 | 1 | 0 | -1 | | | 0 | | 0 | | 1 | | 0 | | 1 | | 5 | Probable |
| HOP-569 | Amoxicillin - clavulanic acid | | Adenitis | | 1 | 2 | 1 | 0 | -1 | | | 0 | | 0 | | 1 | | 0 | | 1 | | 5 | Probable |
| HOP-537 | Azithromycine | | Bronchitis | | 1 | 2 | 1 | 0 | -1 | | | 0 | | 0 | | 1 | | 0 | | 1 | | 5 | Probable |
| HOP-533 | Clarithromycine | | Bronchitis | | 1 | 2 | 0 | 0 | -1 | | | 0 | | 0 | | 0 | | 0 | | 1 | | 3 | Possible |
| HOP-553 | Clindamycine | | Infection | | 0 | 2 | 1 | 0 | -1 | | | 0 | | 0 | | 1 | | 0 | | 1 | | 4 | Possible |
| HOP-574 | Metronidazole | | Infectious colitis | | 1 | 2 | 1 | 0 | -1 | | | 0 | | 0 | | 1 | | 0 | | 1 | | 5 | Probable |
| HOP-532 | Minocycline | | Rosacea | | 1 | 2 | 1 | 0 | -1 | | | 0 | | 0 | | 1 | | 0 | | 1 | | 5 | Probable |
| HOP-505 | Trimethoprime-Sulfamethoxazole | | Cystitis | | 1 | 2 | 1 | 0 | -1 | | | 0 | | 0 | | 1 | | 0 | | 1 | | 5 | Probable |
| HOP-508 | Trimethoprime-Sulfamethoxazole | | Cystitis | | 1 | 2 | 1 | 2 | -1 | | | 0 | | 0 | | 1 | | 0 | | 1 | | 7 | Probable |
| ***Antidiarrheals, intestinal antiinflammatory / antiinfective agents*** | | | | |  |  |  |  |  | | |  | |  | |  | |  | |  | |  |  |
| HOP-508 | Mesalazine | | Ulcerative colitis | | 1 | 2 | 1 | 0 | -1 | | | 0 | | 0 | | 1 | | 0 | | 1 | | 5 | Probable |
| HOP-522 | Mesalazine | | Ulcerative colitis | | 1 | 2 | 1 | 0 | -1 | | | 0 | | 0 | | 1 | | 0 | | 1 | | 5 | Probable |
| HOP-559 | Mesalazine | | Ulcerative colitis | | 1 | 2 | 1 | 0 | -1 | | | 0 | | 0 | | 1 | | 0 | | 1 | | 5 | Probable |
| HOP-605 | Sulfasalazine | | Juvenile Rheumatoid arthritis | | 1 | 2 | 1 | 0 | -1 | | | 0 | | 0 | | 1 | | 0 | | 1 | | 5 | Probable |
| ***Antiinflammatory and antirheumatic products*** | | | |  | | | | | | | | | | | | | | | | | | | |
| HOP-508 | Ibuprofen | | Inflammation | | 1 | 2 | 1 | 0 | | -1 | | 0 | | 0 | | 1 | | 0 | | 1 | | 5 | Probable |
| HOP-514 | Diclofenac | | Inflammation | | 1 | 2 | 1 | 0 | | -1 | | 0 | | 0 | | 1 | | 0 | | 1 | | 5 | Probable |
| HOP-570 | Celecoxib | | Inflammation | | 1 | 2 | 1 | 0 | | -1 | | 0 | | 0 | | 1 | | 0 | | 1 | | 5 | Probable |
| HOP-584 | Naproxen | | Inflammation | | 1 | 2 | 1 | 0 | | -1 | | 0 | | 0 | | 1 | | 0 | | 1 | | 5 | Probable |
| ***Lipid modifying agents*** | | |  | |  |  |  |  | |  | |  | |  | |  | |  | |  | |  |  |
| HOP-530 | Atorvastatin | | Hypercholesterolemia | | 1 | 2 | 1 | 0 | | -1 | | 0 | | 0 | | 1 | | 0 | | 1 | | 5 | Probable |
| HOP-534 | Atorvastatin | | Hypercholesterolemia | | 1 | 2 | 1 | 0 | | -1 | | 0 | | 0 | | 1 | | 0 | | 1 | | 5 | Probable |
| HOP-558 | Atorvastatin | | Hypercholesterolemia | | 1 | 2 | 1 | 2 | | -1 | | 0 | | 0 | | 1 | | 0 | | 1 | | 7 | Probable* |
| HOP-558 | Atorvastatin | | Hypercholesterolemia | | 1 | 2 | 1 | 2 | | -1 | | 0 | | 0 | | 1 | | 1 | | 1 | | 8 | Probable* |
| HOP-576 | Atorvastatin | | Hypercholesterolemia | | 1 | 2 | 1 | 0 | | -1 | | 0 | | 0 | | 1 | | 1 | | 1 | | 6 | Probable |
| HOP-589 | Atorvastatin | | Hypercholesterolemia | | 1 | 2 | 1 | 0 | | -1 | | 0 | | 0 | | 1 | | 0 | | 1 | | 5 | Probable |
| HOP-600 | Ezetimibe | | Hypercholesterolemia | | 1 | 2 | 1 | 0 | | -1 | | 0 | | 0 | | 1 | | 0 | | 1 | | 5 | Probable |
| HOP-576 | Rosuvastatin | | Hypercholesterolemia | | 1 | 2 | 1 | 2 | | -1 | | 0 | | 0 | | 1 | | 1 | | 1 | | 8 | Probable* |
| HOP-576 | Rosuvastatin | | Hypercholesterolemia | | 1 | 2 | 1 | 2 | | -1 | | 0 | | 0 | | 1 | | 1 | | 1 | | 8 | Probable* |
| HOP-583 | Rosuvastatin | | Hypercholesterolemia | | 1 | 2 | 1 | 0 | | -1 | | 0 | | 0 | | 1 | | 0 | | 1 | | 5 | Probable |
| HOP-528 | Rosuvastatin | | Hypercholesterolemia | | 1 | 2 | 1 | 0 | | -1 | | 0 | | 0 | | 1 | | 0 | | 1 | | 5 | Probable |
| HOP-535 | Simvastatin | | Hypercholesterolemia | | 1 | 2 | 1 | 2 | | -1 | | 0 | | 0 | | 1 | | 1 | | 1 | | 8 | Probable |
| ***Antiepileptics*** | | |  | |  |  |  |  | |  | |  | |  | |  | |  | |  | |  |  |
| HOP-575 | Carbamazepine | | Familial neuropathy | | 1 | 2 | 1 | 0 | | -1 | | 0 | | 0 | | 1 | | 0 | | 1 | | 5 | Probable |
| HOP-511 | Phenytoin | | Nausea | | 1 | 2 | 1 | 0 | | -1 | | 0 | | 0 | | 1 | | 0 | | 1 | | 5 | Probable |
| ***Agents acting on the renin-angiotensin system*** | | | |  | | | | | | | | | | | | | | | | | | | |
| HOP-519 | Losartan-Hydrochlorothiazide | | High blood pressure | | 1 | 2 | 1 | 0 | | | -1 | | 0 | | 0 | | 1 | | 0 | | 1 | 5 | Probable |
| HOP-526 | Perindopril | | High blood pressure | | 1 | 2 | 1 | 0 | | | 2 | | 0 | | 0 | | 1 | | 0 | | 1 | 8 | Probable |
| ***Cough and cold preparations*** | | |  | |  |  |  |  | | |  | |  | |  | |  | |  | |  |  |  |
| HOP-541 | Codeine | | Pain | | 1 | 2 | 1 | 0 | | | -1 | | 0 | | 0 | | 1 | | 0 | | 1 | 5 | Probable |
| HOP-548 | Codeine | | Pain | | 1 | 2 | 1 | 0 | | | -1 | | 0 | | 0 | | 1 | | 0 | | 1 | 5 | Probable |
| HOP-555 | Codeine | | Pain | | 1 | 2 | 1 | 0 | | | -1 | | 0 | | 0 | | 1 | | 0 | | 1 | 5 | Probable |
| HOP-595 | Codeine | | Pain | | 1 | 2 | 1 | 0 | | | -1 | | 0 | | 0 | | 1 | | 0 | | 1 | 5 | Probable |
| ***Psychoanaleptics*** | | |  | |  |  |  |  | | |  | |  | |  | |  | |  | |  |  |  |
| HOP-593 | Sertraline | | Eating disorders | | 1 | 2 | 1 | 0 | | | -1 | | 0 | | 0 | | 1 | | 0 | | 1 | 5 | Probable |
| HOP-542 | Venlafaxine | | Depression | | 1 | 2 | 0 | -1 | | | -1 | | 0 | | 0 | | 1 | | 0 | | 1 | 3 | Possible |
| HOP-546 | Venlafaxine | | Depression | | 1 | 2 | 1 | 0 | | | -1 | | 0 | | 0 | | 1 | | 0 | | 1 | 5 | Probable |
| ***Corticosteroids for systemic use*** | | |  | |  |  |  |  | | |  | |  | |  | |  | |  | |  |  |  |
| HOP-577 | Dexamethasone | | Hiatus hernia | | 1 | 2 | 1 | 0 | | | -1 | | 0 | | 0 | | 0 | | 0 | | 1 | 4 | Possible |
| HOP-598 | Dexamethasone | | Inflammation | | 1 | 2 | 1 | 0 | | | -1 | | 0 | | 0 | | 1 | | 0 | | 1 | 5 | Probable |
| HOP-579 | Triamcinolone | | Rotator cuff tear | | 1 | 2 | 0 | 2 | | | -1 | | 0 | | 0 | | 1 | | 1 | | 1 | 7 | Probable* |
| HOP-579 | Triamcinolone | | Rotator cuff tear | | 1 | 2 | 0 | 2 | | | -1 | | 0 | | 0 | | 1 | | 1 | | 1 | 7 | Probable* |
| HOP-579 | Triamcinolone | | Rotator cuff tear | | 1 | 2 | 0 | 2 | | | -1 | | 0 | | 0 | | 1 | | 1 | | 1 | 7 | Probable* |
| ***Analgesics*** | |  |  | |  |  |  |  | | |  | |  | |  | |  | |  | |  |  |  |
| HOP-597 | Immediate-release morphine | | Pain | | 0 | 2 | 1 | 0 | | | -1 | | 0 | | 0 | | 1 | | 0 | | 1 | 4 | Possible |
| HOP-548 | Morphine | | Pain | | 0 | 2 | 1 | 0 | | | -1 | | 0 | | 0 | | 1 | | 1 | | 1 | 5 | Probable |
| ***Psycholeptics*** | | |  | |  |  |  |  | | |  | |  | |  | |  | |  | |  |  |  |
| HOP-566 | Lurasidone | | Bipolar disorder | | 1 | 2 | 1 | 0 | | | -1 | | 0 | | 0 | | 1 | | 0 | | 1 | 5 | Probable |
| ***Endocrine therapy*** | | |  | |  |  |  |  | | |  | |  | |  | |  | |  | |  |  |  |
| HOP-550 | Tamoxifen | | Breast cancer | | 1 | 2 | 1 | 0 | | | -1 | | 0 | | 0 | | 1 | | 0 | | 1 | 5 | Probable |
| ***Antimycobacterials*** | | |  | |  |  |  |  | | |  | |  | |  | |  | |  | |  |  |  |
| HOP-582 | Rifampin | | Infection | | 1 | 2 | 1 | 0 | | | -1 | | 0 | | 0 | | 1 | | 0 | | 1 | 5 | Probable |
| ^1^ The Naranjo scale is used to estimate the probability of adverse drug reactions. A pointing system of ten questions provides a probability | | | | | | | | | | | | | | | | | | | | | | | |
| category from the total score (definite ≥ 9; Probable 5 to 8; Possible 1 to 4; doubtful ≤ 0) | | | | | | | | | | | | | | | | | | | | | | | |
| The asterisk (*) means that the adverse drug reaction is certain since cases are rechallenged | | | | | | | | | | | | | | | | | | | | | | | |
